# Validation of QTL mapping and transcriptome profiling for identification of candidate genes associated with nitrogen stress tolerance in sorghum

**DOI:** 10.1186/s12870-017-1064-9

**Published:** 2017-07-11

**Authors:** Malleswari Gelli, Anji Reddy Konda, Kan Liu, Chi Zhang, Thomas E. Clemente, David R. Holding, Ismail M. Dweikat

**Affiliations:** 1 Department of Agronomy and Horticulture, University of Nebraska, Lincoln, NE 68583 USA; 2 Department of Biochemistry, University of Nebraska, Lincoln, NE 68588 USA; 3 Center for Plant Science Innovation, University of Nebraska, Lincoln, NE 68588 USA; 4 School of Biological Sciences, University of Nebraska, Lincoln, NE 68588 USA

**Keywords:** Sorghum, Nitrogen use efficiency (NUE), N-stress tolerance, Genotyping-by-sequencing (GBS), QTL mapping, QTL validation, Agronomic traits, Illumina RNA-seq, Differentially expressed gene transcripts, Candidate genes, Marker assisted selection (MAS)

## Abstract

**Background:**

Quantitative trait loci (QTLs) detected in one mapping population may not be detected in other mapping populations at all the time. Therefore, before being used for marker assisted breeding, QTLs need to be validated in different environments and/or genetic backgrounds to rule out statistical anomalies. In this regard, we mapped the QTLs controlling various agronomic traits in a recombinant inbred line (RIL) population in response to Nitrogen (N) stress and validated these with the reported QTLs in our earlier study to find the stable and consistent QTLs across populations. Also, with Illumina RNA-sequencing we checked the differential expression of gene (DEG) transcripts between parents and pools of RILs with high and low nitrogen use efficiency (NUE) and overlaid these DEGs on to the common validated QTLs to find candidate genes associated with N-stress tolerance in sorghum.

**Results:**

An F_7_ RIL population derived from a cross between CK60 (N-stress sensitive) and San Chi San (N-stress tolerant) inbred sorghum lines was used to map QTLs for 11 agronomic traits tested under different N-levels. Composite interval mapping analysis detected a total of 32 QTLs for 11 agronomic traits. Validation of these QTLs revealed that of the detected, nine QTLs from this population were consistent with the reported QTLs in earlier study using CK60/China17 RIL population. The validated QTLs were located on chromosomes 1, 6, 7, 8, and 9. In addition, root transcriptomic profiling detected 55 and 20 differentially expressed gene (DEG) transcripts between parents and pools of RILs with high and low NUE respectively. Also, overlay of these DEG transcripts on to the validated QTLs found candidate genes transcripts for NUE and also showed the expected differential expression. For example, DEG transcripts encoding *Lysine histidine transporter 1* (LHT1) had abundant expression in San Chi San and the tolerant RIL pool, whereas DEG transcripts encoding seed storage albumin, transcription factor IIIC (TFIIIC) and dwarfing gene (*DW2*) encoding multidrug resistance-associated protein-9 homolog showed abundant expression in CK60 parent, similar to earlier study.

**Conclusions:**

The validated QTLs among different mapping populations would be the most reliable and stable QTLs across germplasm. The DEG transcripts found in the validated QTL regions will serve as future candidate genes for enhancing NUE in sorghum using molecular approaches.

**Electronic supplementary material:**

The online version of this article (doi:10.1186/s12870-017-1064-9) contains supplementary material, which is available to authorized users.

## Background

Nitrogen (N) is the most abundant and highly required mineral nutrient taken up by plants from the soil [1] and is often a limiting factor for plant growth and development [2]. Nitrogen is an important constituent of amino acids, proteins, hormones, chlorophyll and comprises 16% of the total plant protein [3]. In the last four decades, crop breeding along with increased use of synthetic nitrogen fertilizers [4] resulted in significant increase in global food production and satiated world hunger [4–6]. However, N fertilizer production consumes ~1% of the world’s total annual energy supply, adding to food production costs [7]. Furthermore, it has been estimated that 50 to 70% of the nitrogen applied to the soil is lost into the atmosphere and through leaching accelerates eutrophication of water ways, acidification of soils [8] and produces greenhouse gases [9]. The heavy reliance on fertilizer application to achieve higher yields has resulted in a greater need for environmental protection measures. Therefore, increasing nitrogen use efficiency (NUE) by developing crops and/or genotypes that yield better with limited N supply is a crucial goal to protect the environment [10, 11] and help towards more sustainable and productive agriculture [12], which has been recognized in a call for “Second Green Revolution”.

Sorghum [*Sorghum bicolor* (L.) Moench] is one of the world’s most important cereal crops and originated from Africa [13]. Currently, sorghum feeds over 500 million people in 98 countries [14] and also used as an important animal feed in many countries [15]. Moreover, sorghum is the important source for grain-based ethanol production in U.S. next to maize and almost 12% of the production was used [16, 17]. Sorghum is C_4_ photosynthetic and is well-adapted to high temperature and limited water environments [14, 18]. Sorghum uses nitrogen, CO_2_ and water more efficiently than maize and is a model crop species for tropical genomes [19]. Like other cereals, sorghum depends heavily on applied nitrogen fertilizers to achieve commercial yields. Sorghum is drought tolerant with its deep root system [20] and developing varieties that yield better with limited N-supply would make it as a suitable crop for cultivation in arid regions.

In higher plants, N-limitation causes adverse effects on crop growth and yield [5]. The phenomenon of nitrogen use efficiency in plants is complex and has been defined as the grain yield [21] or fresh/dry matter produced [5] per unit of nitrogen available in the soil. Plant responsiveness to nitrogen availability depends on both genotype and the interaction of genotype with the level of N fertilization [22]. Under high nitrogen input conditions, variation in NUE results mainly from differences in N uptake. In contrast, under low N conditions, variation in NUE is determined largely by changes in N remobilization and utilization efficiency [23]. Some genetic variability exists for nitrogen use efficiency and its components, N uptake and utilization and has been reported in rice [24], wheat [25], sorghum [26], and maize [27–29]. Such variability is a valuable resource to help understand the genetic basis of nitrogen use and its exploitation. Several QTL mapping studies targeting various traits associated with NUE and N-stress tolerance have been reported in barley [30–32], maize [33–35], rice [36–38], wheat [39–44], and *Arabidopsis* [45]. To date, in sorghum only a single study has been reported to map the QTLs controlling various agronomic traits tested under N-stress using a bi-parental (CK60/China17) mapping population [46].

In crop breeding, the assumption earlier was that most markers associated with QTLs from preliminary mapping studies were directly useful in marker assisted selection (MAS) for enhancing breeding efficiency. However, results from QTL mapping may vary among studies because of the use of different backgrounds, environments, and sampling variation [47]. However, in recent years it has become widely accepted that QTL confirmation/validation and/or fine (high resolution) mapping may be required [48] in different environments and/or genetic backgrounds before being used for marker assisted breeding. In the past, different approaches have been used for validating the QTLs. Of these, one approach is to map QTLs in early generations of the population and get validated in advanced breeding generations from the same cross. For example; in rice, Wickneswari et al. [49] validated the QTLs for multiple agronomic traits detected in BC_2_F_2_ generation in advanced breeding generations such as BC_2_F_5_ derived from the same parents. Similarly, in sweet sorghum Wang et al. [50] validated the partially dominant QTLs mapped earlier [51] for plant height, fresh stem and leaf weight, juice weight and brix in F_2_ and F_2:3_ by developing RILs from the same parents. Such validation confirms the beneficial effect of the QTLs detected in early generations and the breeding lines carrying these significant QTLs could be considered for direct use in varietal development or as pre-breeding material to develop new cultivars. Another approach to validate genetic markers across populations is to develop multiple mapping populations and perform interval mapping on each to identify QTLs which are common between the populations. Haussmann et al. [52] used this approach and mapped QTLs for stay green in two RIL populations of sorghum. Similarly, in sorghum [53] validated important stay green QTLs mapped in the past using a new RIL population derived from a cross between M35-1 and B35. In Barley Zhou et al. [54] mapped QTLs for kernel length in a bi-parental RIL population and validation in a different RIL population mapped two major QTLs.

Linkage-based QTL studies do not usually provide a framework to distinguish among candidate genes without further fine-mapping [55]. In recent years various high throughput sequencing strategies have been developed for genome re-sequencing [56–58], resulting in a large amount of single nucleotide polymorphism (SNP) data being generated for sorghum [59, 60]. To enhance the utility of sorghum SNP data, Luo et al. [61] developed a web-based large-scale genome variation database (SorGSD, http://sorgsd.big.ac.cn), containing ~62.9 million SNPs from a diverse panel of 48 sorghum lines. These SNPs will be a valuable resource for genetic and breeding studies for efficient discovery of key QTLs or genes relevant to important traits. Increasingly, there has been also a desire to identify the candidate genes underlying the QTLs responsible for traits of interest. The most common method to define candidates underlying a QTL is to search for physically-proximate genes with annotations or gene ontology reflecting the trait of interest [62]. Availability of the sorghum reference genome sequence [63] and emergence of next gen sequencing technologies provide large amounts of sequence information that can be annotated to examine the role of specific genes and transcripts associated with traits of interest. For example, using the whole genome sequencing data generated on diverse sorghum lines, Massel et al. [64] assessed the genetic diversity across 230 fully sequenced genes putatively involved in N-uptake and utilization to find gene targets to improve NUE in sorghum and other cereals. In addition, the use of next-generation sequencing approaches such as RNA-seq was utilized to profile transcriptomes and detect differentially expressed genes (DEGs) in sorghum for nitrogen, osmosis and cold stresses [65–67]. Given that we have been able to dissect genetic architecture of stress tolerance into several chromosomal loci by QTL mapping, the combined use of the QTL mapping with transcriptome profiling represents a practical solution to further refine the mapping resolution and identify potential candidate genes [46, 68].

The objectives of this study were: 1) to map QTLs for various agronomic traits tested under different N-treatments in a sorghum RIL population of a cross (CK60/San Chi San) with contrasting N-stress tolerance. 2) to validate QTLs from this study with the reported QTLs of our earlier study with different RIL population and to evaluate the consistency and stability of the QTLs. 3) to identify the DEG transcripts between parents and bulks of RILs with low and high NUE and overlay these DEGs on to the validated QTLs across mapping populations to identify candidate gene transcripts associated with N-stress response.

## Methods

### Development of the RIL mapping population

An F_7_ RIL population consisting of 208 individuals was developed from the cross between inbred lines CK60 and San Chi San and was used for this study. CK60 is a short, photoperiod-sensitive, late-maturing U.S. grown public sorghum inbred line and an inefficient nitrogen user. San Chi San is a tall, photoperiod-insensitive, early-maturing Chinese sorghum inbred line and an efficient nitrogen user and seed was provided by Jerry Maranville (University of Nebraska, Lincoln). Green house evaluations of CK60 and San Chi San genotypes under low and high N showed that CK60 retains higher chlorophyll content but San Chi San had higher carbon exchange rates under N-stress [69]. The seedlings of San Chi San had greater root and shoot mass compared to CK60 under both low N and normal N conditions [65]. Each of the RILs was derived from a single F_2_ plant following the single seed descent method until the F_7_ generation [70].

### Field trails

The F_7_ RIL population and two parental lines (CK60 and San Chi San) were evaluated in an alpha lattice incomplete block design at University of Nebraska-Lincoln experimental farms. The experiments were conducted in the field with two different nitrogen treatments such as normal N (NN) and low N (LN), with two independent replicates for each N-treatment during summer of years 2011 and 2012. The individuals were randomized and planted in 15 blocks per replication. As presented in our earlier study [46], soil testing results showed that there is no residual ammonium acetate left in both low and normal N fields. However, these fields have various extents of other minerals including nitrate, P, K, Ca, Mg and Na. The low N fields were provided with 0 kg. ha^−1^ synthetic fertilizer and was rotated prior with oats and maize to deplete the residual N. This field had not received any nitrogen fertilizer since 1986. The normal N fields were provided with 100 kg. ha^−1^ anhydrous ammonia fertilizer and rotated prior with soybeans to supplement the nitrogen. The experimental units were planted in five meters long rows with 0.75 m spacing with a density of 50 seeds for RILs and parents. All entries were planted on the same day in conventionally-tilled plots and maintained under rain fed conditions.

### Evaluation of important agronomic traits

Three plants were randomly selected for each genotype and tagged for phenotypic evaluation of 11 agronomic traits. The measured phenotypes include:

1. Leaf chlorophyll content at vegetative stage (Chl1), measured before flowering on the 3rd leaf from top with a portable chlorophyll meter model SPAD-502 (*Konica-Minolta,* Co. *Ltd.,* Tokyo, Japan). Measurements taken at three different places away from the midrib on third leaf from top and were averaged for each plot.
2. Leaf chlorophyll content at flowering (Chl2), measured during flowering similar to Chl1.
3. Leaf chlorophyll content at maturity (Chl3), measured at maturity similar to Chl1 and Chl2. Haussmann et al. [52] described that the upper six leaves are a good source for measuring the greenness of leaves since they are photosynthetically active at anthesis and contribute nutrients to the grain [71].
4. Plant height (PH), measured from the base of the plant to tip of the panicle/head in centimeters at physiological maturity.
5. Days to anthesis (AD), counted the number of days from planting to 50% of plants in a plot reached flowering stage.
6. At physiological maturity, the three tagged plants were harvested manually. Panicles were separated from rest of the plant and weighed to get an average fresh panicle weight. Then, panicles were dried for 10 to 14 d in hot air dryer and weighed to get an average dry panicle weights. Head moisture contents (MC2), was calculated as the % difference between fresh and dry weight of the three panicles.
7. After harvesting, rest of the vegetative tissues (without panicles) of the three plants was weighed to get fresh biomass weight and dried in hot air oven for ten days to get the dry weights. Stover moisture contents (MC1) was calculated as the % difference between fresh and dry weights of total aboveground vegetative tissues.
8. Biomass yield (BY, t. ha^−1^), measured average dry weight (g) of the total above ground vegetative tissues from three randomly selected plants and expressed in t. ha^−1^ using the conversion ((biomass weight (g) /plot area (m^2^)) *(1 ton/100000 g) *(10,000 m^2^/ha)).
9. Grain yield (GY, t. ha^−1^), measured average grain weight (g) from three selected plants after threshing and expressed in t. ha^−1^ using the conversion ((grain weight (g) /plot area (m^2^)) *(1 ton/100000 g) *(10000m^2^/ha)).
10. 1000 grain weight (TGW) in grams.
11. Grain-to-stover ratio (GS, %), measured as (grain yield/biomass yield) ^*^100.

All the phenotypes were measured from low N and normal N fields from two replications in each year. In summary, the phenotypes were classified into three groups, chlorophyll contents (Chl1, Chl2, and Chl3), morphological traits (PH, AD, MC1, and MC2), and yield-related traits (BY, GY, TGW and GS).

### Phenotypic data analysis

The statistical software SAS 9.2 (Statistical Analysis Systems Institute Inc., Cary, N.C.) was used for analyzing the phenotypic data. Trait variances were portioned into individual effects using the following statistical model: *Y*_*ijkl*_ *= μ + g*_*i*_ *+ t*_*j*_ *+ g*_*i*_ *t*_*j*_ *+ r*_*k*_ *+ b*_*l(k, j)*_ *+ e*_*ijkl*_, where *Y*_*ijkl*_ is the response of *i* ^th^ genotype in *l* ^th^ block of *k* ^th^ replication in *t* ^th^ nitrogen treatment, *μ* is the grand mean of the phenotype, *g*_*i*_ is the genotype or line effect, *t*_*j*_ is the nitrogen treatment effect, *g*_*i*_ *t*_*j*_ is the genotype by nitrogen treatment interaction, *r*_*k*_ is the replications effect, *b*_*l(k,j)*_ is the block effect in replicate within nitrogen treatment and e_ijk_ is the residual. Analysis of variance (ANOVA) for eleven traits was performed for nitrogen treatments in each year separately using the PROC MIXED procedure [72] where the genotype effect was considered as fixed, replications and blocks effect as random. Genotype x N-treatment interaction was mainly associated with differences in magnitude of effects between years (data not shown). So, the phenotypic data from 2011 and 2012 were pooled to obtain a single trait value for each N-treatment (Comb-LN and Comb-NN) [33]. ANOVA was performed on pooled data by considering genotype, N-treatments and genotype by N treatment interaction (GxE) effects were fixed and replications within N treatments, blocks within replications and N-treatments were random. Narrow-sense heritability with standard error was estimated using the PROC MIXED procedure of SAS version 9.2. For the heritability estimates, parental lines data were excluded, and estimates followed a method described by Holland et al. [73]. Pearson’s correlation coefficients between traits were calculated for the least square genotype means using the PROC CORR procedure of SAS. The RIL trait data were subjected to a normality test using PROC UNIVARIATE to determine its suitability for QTL analysis.

### SNP discovery using genotyping-by-sequencing

Total genomic DNA of the RILs and their parents were isolated from leaf tissues using a DNeasy Plant Mini Kit (Qiagen). DNA quality was assessed by 260/280 nm absorbance ratios with a Biophotometer 6131 (Eppendorf, Hauppauge, NY). DNA was quantified using the QuantiFluor dsDNA labeling system (Promega, Madison, WI) with a TBS Mini-Fluorometer (Turner Biosystems, Sunnyvale, CA). We followed the ‘Genotyping by Sequencing’ (GBS) method of Elshire et al. [56] to generate ApeKI-associated DNA fragments for sequencing on the Illumina HiSeq® 2000 platform. DNA (500 ng) from each sample was digested with ApeKI (New England Bio-labs, Ipswich, MA), a type II restriction endonuclease that recognizes a degenerate 5 bp sequence (5′-GCWGC) and creates 5′ overhangs. Adapters with specific barcodes [56] were then ligated to the restriction-digested overhanging sequences using T_4_ ligase. A set of 96 DNA samples, each sample with a different barcode adapter, were combined and purified (Quick PCR Purification Kit; Qiagen, Valencia, CA) according to the manufacturer’s instructions. DNA fragments containing ligated adapters were amplified with primers containing complementary sequences for each adapter. PCR products were then purified and diluted for sequencing [56]. Single-end, 100 bp reads were collected for one 48- or 96-plex library per flow cell channel on a Genome Analyzer IIx (GAIIx; Illumina, Inc., San Diego, CA) [74] at Cornell University Biotechnology Resource Center, USA. The 86 bp raw reads from GAIIx were filtered [56] and aligned to the *Sorghum bicolor* reference genomes version 1.4 and 2.1 by downloading the genome sequences from http://genome.jgi.doe.gov/pages/dynamicOrganismDownload.jsf?organism=Sbicolor. The genotypes of the population were determined based on the procedure described by Elshire et al. [56].

### Genetic map construction

SNP data were converted to the ‘a, h, b’ codes with the female parent conferring the ‘a’ genotype, male parent with ‘b’ and heterozygous with ‘h’ alleles. The bi-allelic GBS markers were checked for polymorphism between the parents. Highly similar markers (>0.95) were excluded from the data set to reduce calculation time. Prior to map construction, all polymorphic SNPs were checked using a chi-square (χ2) test for the goodness of fit against a 1:1 segregation ratio at the 0.05 probability level. SNPs with >70% missing data were removed from the data set. A total of 844 polymorphic SNPs were selected and used for constructing linkage maps using Mapmaker/EXP 3.0 along with IciMapping (Inclusive composite interval mapping) v3.2 [75]. The genetic distance (cM) was calculated using the Kosambi mapping function. Out of these polymorphic SNPs used for genetic map construction, the final map consists of 833 SNPs. The genetic map spanned a length of 1527 cM and distributed across the 10 chromosomes of sorghum (Additional file 1). The average genetic distance between adjacent markers was 1.8 cM and linkage groups were assigned to ten chromosomes.

### Quantitative trait loci analysis

The QTL analysis was performed for the trait means obtained from each year (2011 and 2012) for normal N and low N treatments (11NN, 12NN, 11LN, 12LN), and for averaged trait means across two years for each N treatment (Comb-NN, Comb-LN) using composite interval mapping (CIM) method of WinQTLcart2.5 [76]. The CIM analysis was run using Model 6 with forward and backward stepwise regression with a probability in and out of 0.1 and with a window size of 10 cM. The walking speed chosen for all traits was 1 cM. Experiment wise significance thresholds (*P* ≤ 0.05) for QTL detection were determined with 1000 permutations. The location of a significant QTL was determined according to its logarithm of odds (LOD) peaks [77]. A 2-LOD support interval was calculated for each QTL to obtain a 95% confidence interval. Adjacent QTLs on the same chromosome for the same trait were considered as different when the support intervals were non-overlapping. The contribution rate (*R* ^2^) was calculated as the percentage of variance explained by each QTL in proportion to the total phenotypic variance. The additive effect of a putative QTL was estimated by half the difference between two homozygous classes. QTLs were named according to McCouch et al. [78] and alphabetic order was used for QTLs on the same chromosome. QTL were classified as *major* if the phenotypic variance explained was larger than 10% and *minor* if variance explained is less than 10% [79]. QTLs with a positive or negative additive effect for a trait imply that the increase in the phenotypic value of the trait is contributed by alleles from CK60 or San Chi San. The graphic representations of QTLs on linkage groups were drawn by MapChart 2.2 software [80]. Flanking marker intervals of the QTLs detected in this study using CK60/San Chi San population were compared with the intervals of the QTLs detected for N-stress tolerance in our earlier study using CK60/China17 population [46], and QTLs with overlapped intervals will be considered as validated QTLs among the two mapping populations.

### Screening of candidate DEG transcripts associated with QTLs for N-stress tolerance.

In our earlier study [65], we identified several DEG transcripts between the transcriptomes of seven sorghum genotypes using Illumina RNA sequencing. Transcriptomes were prepared from root tissues of three-week seedlings grown under N-stress from four N-stress tolerant (China17, San Chi San, KS78 and high NUE RIL bulk) and three sensitive (CK60, BTx623 and low NUE RIL bulk) genotypes of sorghum. RIL bulks with high and low NUE were made by mixing the equal quantity of RNA extracted from root tissues of the five best performing RILs and five poor performing RILs of CK60/San Chi San population respectively. These RILs were selected based on their biomass yield tested under low N field conditions. In this study, we used the RNA-seq data generated earlier [65] in order to find DEG transcripts between CK60 and San Chi San, bulks of RILs with high and low NUE. Pair-wise comparison was made between the transcriptomes of CK60 and San Chi San, bulks of RILs with high and low NUE to detect DEG transcripts. For false discovery rate (FDR), the Benjamini and Hochberg [81] algorithm with a cutoff setting of <2 was applied. Genes with *P* value ≤0.001 and the cutoff of log_2_-fold value >1 (2-fold absolute value) were considered to be differentially expressed between the genotypes. To compare the differential expression of gene transcripts between CK60 vs. San Chi San and bulks of RILs with high vs. low NUE, we took CK60 and the low NUE RIL bulk as the baseline controls, respectively. Then, the DEG transcripts between parents and RIL bulks were overlaid to the QTL confidence intervals using physical positions to identify candidate DEG transcripts associated with QTLs for agronomic traits expressed under the N-stress.

## Results

### Phenotypic evaluation of the mapping population

Trait mean values of parents CK60 and San Chi San and their RIL population evaluated for 11 agronomic traits for two years in normal N (NN) and low N (LN) environments were shown in Table 1. Under LN environment, the two parental lines differed for most of the traits except chlorophyll content measured at flowering (Chl2). The mean chlorophyll content was higher at flowering (Chl2) than at vegetative (Chl1) and maturity stages (Chl3) under both N-regimes. CK60 has higher chlorophyll content at all the three stages of plant growth under NN and high chlorophyll content at maturity (Chl3) under LN conditions. Days to anthesis was affected by N-condition as low N delayed maturity in both parents. CK60 matured late and had higher stover and head moisture contents than San Chi San under both N conditions. San Chi San was taller, had higher biomass yield, grain yield, thousand grain weight and grain-to-stover ratio compared to CK60 under NN and LN. The mean plant height, biomass and grain yield of the RILs were reduced under LN compared to NN, which is a possible result of limitation in supply of photosynthetic products [27]. The grain-to-stover ratio of CK60 was decreased to almost half in LN compared to NN, while no major change was observed in San Chi San. The averages of thousand grain weight, grain-to-stover ratio and stover moisture content of the RILs remained the same under both N conditions. However, the averages of grain moisture content and days to anthesis in RIL population increased under LN conditions. A wide range of variation for the investigated traits in RIL population (Table 1), normal phenotypic distribution and transgressive segregations (data not shown) suggested a polygenic inheritance of the traits in both N levels. Analysis of variance (ANOVA) performed on the pooled average phenotypic data from two years for each N-treatment (across two Normal-N and Low-N) was shown in Table 2. The calculated F values of traits showed the presence of significant differences (*P* < 0.05) among the RILs for all traits except stover moisture content across two NN. Highly significant N-treatment and genotype x N-treatment interaction effects were observed for most of the traits except grain-to-stover ratio under NN. Genotype variance was greater than genotype x N-treatment interaction variance for most traits (Table 2), suggesting a possibility for detection of significant QTLs that govern nitrogen use efficiency [34]. The estimated narrow sense heritability (*h* ^*2*^) values with standard errors for all the traits were moderate to high (Table 2). The *h* ^*2*^ values ranged from 0.18 (SE = 0.15) for chlorophyll content measured at maturity (Chl3) to 0.46 (SE = 0.08) for days to anthesis under NN. Under LN conditions, *h* ^*2*^ values ranged from 0.36 (SE = 0.1) for grain yield to 0.73 (SE = 0.04) for days to anthesis.

**Table 1:** Mean phenotypic values of parental lines (CK60, San Chi San), min, max, mean phenotypic values and standard deviation of RILs for different agronomic traits measured across two Normal-N and two Low-N treatments

Nitrogen treatment	Source of variation	Chl1	Chl2	Chl3	PH	AD	MC1	MC2	BY	GY	TGW	GS
Normal-N	Ck60	49.9	55.6	53.6	99	71.5	68.6	24.8	7.69	2.89	20.3	0.62
	San Chi San	46.6	52.7	48.3	150	66.3	65	19.5	14.6	6.25	31.6	0.76
	Min	39	42.3	27.6	77	55.1	57.8	13.4	4.21	0.79	17.4	0.16
	Max	58.1	64.5	63.6	220	80.1	76	52.1	21.2	7.32	30.9	2.88
	Mean	47.1	52.8	48.6	133	66	66.9	23.3	10.9	3.54	24.4	0.58
	Std	3.08	3.62	4.68	25.4	3.34	2.8	5.06	3.14	1.42	2.64	0.27
Low-N	Ck60	31.5	36.6	37.3	78	107	66.3	45.1	3.27	0.88	17.4	0.37
	San Chi San	33.2	37.3	25.8	123	84.7	61.7	29.2	6.07	3.08	26.8	0.66
	Min	26.4	26.6	18.7	62.1	64.8	57.2	17.8	2.82	0.04	14.2	0.04
	Max	40.6	47.6	45.4	170	110	72.2	60.2	12.6	4.99	31.3	1.06
	Mean	33.2	36.8	32	110	82.5	64.9	31.4	6.06	1.96	22.7	0.49
	Std	2.73	3.78	4.29	23.8	8.5	3.06	7.84	1.95	0.89	3.36	0.19

Nitrogen treatments: Normal-N and Low-N fields, trait values are averaged over two years (2011 and 2012) under NN and LN treatments respectively. *Chl1, Chl2, Chl3* chlorophyll contents at (vegetative, anthesis, and maturity) stages, *PH* plant height (cm), *AD* days to anthesis, *MC1*% stover moisture content, *MC2*% head moisture content, *BY* biomass yield (t. ha^−1^), *GY* grain yield (t. ha^−1^), *TGW*, thousand grain weight (g), *GS*, grain-to-stover ratio (%)

**Table 2:** ANOVA results, narrow sense heritability estimates with standard error for the traits measured across two normal-N and two low-N conditions

Category	Source of variation	Df	Chl1	Chl2	Chl3	PH	AD	MC1	MC2	BY	GY	TGW	GS
Normal-N													
	Line	207	33.2**	41.9***	62.8**	1985***	34.5***	26***	86.3	32.6**	6.97**	23*	0.24*
	Env	1	458	851**	9218***	17,843	19309***	1628	9598	8.5	11.3	13674**	0.36
	Rep(Env)	2	104.7*	33.8	4.64	18042***	34.3**	636***	6792***	4.35	0.87	208***	0.58*
	Blk(Env*Rep)	54	19.5***	15.1**	38.5**	433.8***	4.72	15.8***	44.8*	15.3**	1.78	7.16	0.14
	Env*Line	172	20.6***	22.4***	41.9***	971***	18.4***	16.4***	69.9***	21.8***	4.7***	17.7***	0.17
	Residual	325	7.78	9.03	23.9	113.4	4.43	6.07	28.71	9.5	1.73	5.44	0.15
	*h* ^*2*^		0.45	0.45	0.18	0.51	0.46	0.39	0.25	0.35	0.37	0.35	0.33
	SE		0.08	0.09	0.15	0.08	0.08	0.09	0.11	0.1	0.09	0.1	0.1
Low-N													
	Line	207	24.4**	49.8**	58.8**	1793***	221***	30***	196***	12**	2.51**	37***	0.11***
	Env	1	2438*	25638**	39857**	984	47395***	428	70.3	942	371**	8075	15.4*
	Rep(Env)	2	140**	115	247**	394	50	327***	8625***	93.2**	4.38	882***	0.5**
	Blk(Env*Rep)	54	14.4	31**	33*	280*	71.3**	16.1**	85.2*	7.71*	1.38**	19.2**	0.04
	Env*Line	172	15.2**	29.5**	35.7**	559***	67.5***	16.3***	102***	7.7***	1.6***	16.4**	0.05***
	Residual	325	11.2	18.6	22.1	192.6	36	9.0	55.5	4.72	0.83	10.5	0.03
	*h* ^*2*^		0.41	0.43	0.43	0.71	0.73	0.51	0.48	0.38	0.36	0.6	0.58
	SE		0.08	0.09	0.15	0.08	0.08	0.09	0.11	0.1	0.09	0.1	0.1

Source of variation: Env, environments (Normal-N and Low-N), Rep, replications; Blk, blocks; df, degrees of freedom; Chl1, Chl2, Chl3, chlorophyll contents (at vegetative, anthesis, and maturity stages); PH, plant height (cm); AD, days to anthesis; MC1, % stover moisture content; MC2, % head moisture content; BY, biomass yield (t. ha^−1^); GY, grain yield (t. ha^−1^); TGW, thousand grain weight (g); GS, grain-to-stover ratio (%). ***P < 0.0001; ** *P* < 0.01; *P < 0.05. h^2^-narrow sense heritability; calculated, SE is standard error

### Correlation between traits

Correlation coefficients among the measured traits were estimated based pooled average line means from 2011 and 2012 years for each N-condition, coefficients are across two normal N and across two low N environments respectively (Table 3). The values on the diagonal represent the trait-specific correlation coefficients between trait values of plants grown at normal N and low N. Significant correlation coefficients were observed for most trait combinations. Grain yield was positively correlated with plant height, biomass yield, thousand grain weight and grain-to-stover ratio under both N-conditions, but negatively correlated with head moisture content in both N levels and with three chlorophyll contents under NN. Highest positive correlation was observed between biomass and grain yield (*r* = 0.80 and *r* = 0.81) under NN and LN conditions respectively. The chlorophyll contents measured at all the three different stages of plant growth were negatively correlated with most of the morphological (plant height, days to anthesis and grain moisture content) and yield traits (biomass yield and thousand grain weight) under both N-levels. Plant height was positively correlated with yield related traits (BY, GY, and TGW) in both N-conditions. Days to anthesis was positively correlated with grain moisture content in both N-conditions, biomass yield, grain yield, thousand grain weight under NN and biomass yield under LN. Positive correlation between days to anthesis and grain moisture content suggests that late maturing lines have higher moisture content in the grains. Both plant height and days to anthesis were negatively correlated with grain-to-stover ratio in both N-levels. Head moisture content was negatively correlated with grain yield and grain-to-stover ratio under NN (*r* = −0.43 and *r* = −0.41) and LN (*r* = −0.27 and *r* = −0.48) conditions respectively.

**Table 3:** Correlation coefficients among the 11 agronomic traits studied across two normal-N and two low-N conditions

	Chl1	Chl2	Chl3	PH	AD	MC1	MC2	BY	GY	TGW	GS
Chl1		0.67***	0.43***	−0.34***	−0.24**	0.056	−0.20**	−0.023	0.081	−0.17*	0.16*
Chl2	0.74***		0.68***	−0.43***	−0.35***	−0.008	−0.23**	−0.029	0.115	−0.071	0.19**
Chl3	0.42***	0.66***		−0.42***	−0.035	0.123	0.103	0.047	0.112	−0.109	0.088
PH	−0.51***	−0.56***	−0.57***		0.049	0.023	−0.20**	0.44***	0.27***	0.41***	−0.088
AD	−0.47***	−0.39***	0.07	0.23**		0.20**	0.67***	0.15*	0.033	−0.29***	−0.16*
MC1	0.14	0.1	0.19**	−0.08	0.05		0.24**	0.056	0.081	−0.023	0.111
MC2	−0.07	−0.12	0.23**	−0.11	0.39***	0.40***		−0.052	−0.27***	−0.39***	−0.48***
BY	−0.43***	−0.40***	−0.30***	0.74***	0.33***	−0.03	−0.07		0.81***	0.18*	0.081
GY	−0.26**	−0.16*	−0.19**	0.54***	0.14*	−0.13	−0.43***	0.80***		0.25**	0.58***
TGW	−0.38***	−0.33***	−0.16*	0.39***	0.25**	−0.05	0.04	0.45***	0.33***		0.23**
GS	0.29***	0.28***	0.107	−0.16*	−0.32***	−0.05	−0.41***	−0.11	0.27***	−0.08	

Correlation coefficients were calculated from the trait values averaged over two years (2011 and 2012). The numbers below the diagonal are correlation coefficients under normal N treatments and numbers above the diagonal are correlation coefficients under low N treatments. Chl1, Chl2, Chl3, chlorophyll contents (at vegetative, anthesis, and maturity stages); PH, plant height (cm); AD, days to anthesis; MC1, % stover moisture content; MC2, % head moisture content; BY, biomass yield (t. ha^−1^); GY, grain yield (t. ha^−1^); TGW, thousand grain weight (g); GS, grain-to-stover ratio (%). ***P < 0.0001; **P < 0.01; *P < 0.05

### QTL analysis using the SNP genetic map

**Fig. 1:**
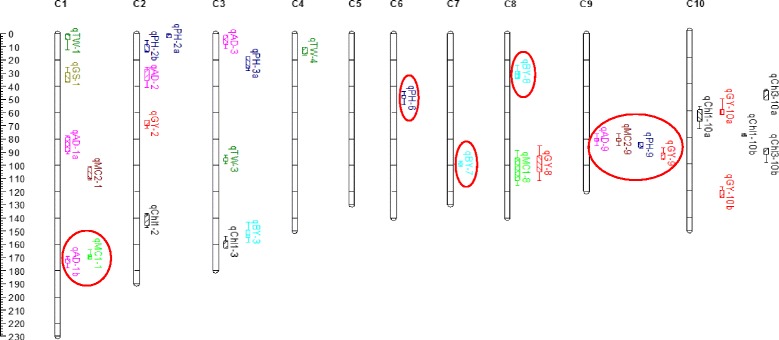
QTL locations for 11 agronomic traits studied in the Ck60/San Chi San RIL population**.** QTLs were represented in different colors for 11 agronomic traits including black for Chl-1, Chl-2, and Chl-3 (chlorophyll contents at vegetative, at anthesis and at maturity stages), blue for PH (plant height, cm), pink for AD (anthesis date, days), lawn green for MC1 (stover moisture content, %), brown for MC2 (head moisture content, %), light blue for BY (biomass yield, t.ha^−1^), red for GY (grain yield, t.ha^−1^), dark green for TW (thousand grain weight, g) and tan for GS (grain to stover ratio, %) on chromosomes C1 to C10. QTLs detected stably across environments are presented by open bars and with different fills for 11LN, 11NN, 12LN and 12NN, comb-NN and comb-LN conditions. Supported intervals for each QTL are indicated by the length of vertical bars. The nine QTLs circled in red were detected in this study and also in our earlier study; these validated QTLs were stable across both mapping populations

QTL analysis was performed to discover chromosomal regions that contribute to the variation observed within the mapping population grown under normal N and low N conditions. Composite interval mapping was conducted on the line mean values from normal and low N conditions in individual years and on pooled average line means from 2011 and 2012 years for each N-condition. The results from QTL analysis for 11 agronomic traits measured under contrasting N-conditions in the RIL population are shown in Fig. 1 and the QTL statistics are summarized in Table 4. Composite interval mapping detected a total of 32 QTLs with LOD thresholds ≥3.0. The R^2^ value is the percent variance explained by each QTL, and is the average performance of plants from NN and LN conditions for individual years and average from two years (2011 and 2012). If a QTL is identified only in one year and one N-level, then the given R^2^ value is specific to that N condition.

**Table 4:** Quantitative trait loci (QTL) detected for 11 agronomic traits studied in CK60/San Chi San RIL population

Trait	QTL name	Environment	Chr	Position (cM)	Flanking markers	Interval (cM)^a^	LOD score	Additive^b^	R^2^ (%)^c^
Chl-1	qChl1-2	12NN	2	141.3	S2_64212168 - S2_66500907	136.5-147.4	3.5	−0.83	6.06
	qChl1-3	12NN	3	159.4	S3_68886972 - S3_71854294	154-163.1	3.6	0.95	8.07
	qChl1-10a	Comb-LN	10	62.8	S10_6734893 - S10_11357374	56.5-73.4	3.8	−1.14	10.7
	qChl1-10b	Comb-LN	10	78.4	S10_13327624 - S10_15068324	77.2-79.3	3.1	0.84	7.46
Chl-3	qChl3-10a	12NN	10	48.2	S10_5936786 - S10_6734839	44.1-52.3	4	−2.9	13.9
	qChl3-10b	12NN	10	91	S10_50340995 - S10_52987481	88.3-98.8	4.9	2.4	11.8
PH	qPH-2a	11NN	2	2.5	S2_1477485 - S2_2197887	0-3.2	3	8.7	7.55
	qPH-2b	11LN	2	10.5	S2_2459689 - S2_3418722	5.4-14	3.2	7	8.04
	qPH-3a	11, 12, Comb- LN	3	21.4	S3_2945750 - S3_4802301	17.4-28.4	3.3	7.9	9.51
	**qPH-6**	**Comb-NN**	**6**	**47.2**	**S6_40065492 - S6_45811964**	**44-54**	**3.8**	**−6.98**	**16.9**
	**qPH-9**	**11NN**	**9**	**85.1**	**S9_50511351 - S9_50956545**	**82.6-87**	**3.3**	**8.9**	**18.0**
AD	qAD-1a	11NN	1	81.2	S1_21655969 - S1_24935643	77.6-91.4	3.4	−1.06	7.06
	**qAD-1b**	**11LN**	**1**	**174**	**S1_58103916 - S1_62800271**	**169-178**	**3.1**	**3.73**	**10.8**
	qAD-2	12NN	2	30.4	S2_4979265 - S2_7797336	25.4-41.1	4.2	−1.45	10.5
	qAD-3	11NN	3	3.1	S3_518323 - S3_2452009	1.4-11.4	5	−1.48	12.8
	**qAD-9**	**11NN, Comb- LN**	**9**	**78.9**	**S9_49568498 - S9_50744450**	**75.9-85**	**5.7**	**1.61**	**14.7**
MC1	**qMC1-1**	**12LN**	**1**	**169.8**	**S1_57571254 - S1_59648859**	**163.9-171.3**	**3**	**0.88**	**10.67**
	qMC1-8	11NN	8	103.3	S8_48291652 - S8_53690396	88.6-115.4	3.3	−0.87	7.48
MC2	qMC2-1	11LN	1	107.7	S1_44806835 - S1_49470786	101.2-111.3	3	−1.82	7.65
	**qMC2-9**	**12LN**	**9**	**78.9**	**S9_49568498 - S9_50744450**	**75.9-85**	**3.1**	**2.92**	**6.76**
BY	qBY-3	12LN	3	151.8	S3_66370259 - S3_70192466	143-158.3	3	0.42	6.81
	**qBY-7**	**11LN, Comb-LN**	**7**	**98.7**	**S7_57890877 - S7_58551650**	**97.1-100.8**	**4.1**	**−1.22**	**11**
	**qBY-8**	**Comb-LN**	**8**	**33.3**	**S8_2528799 - S8_4381062**	**23.9-34.1**	**3.9**	**−0.75**	**10.0**
GY	qGY-2	12NN, Comb-NN	2	65.9	S2_13864242 - S2_19506339	65.6-71.9	3.1	−0.4	7.48
	qGY-8	11LN	8	102.3	S8_48291652 - S8_53253482	85.4-111.6	3.6	0.37	6.63
	**qGY-9**	**Comb-NN**	**9**	**90.6**	**S9_50744450 - S9_51382632**	**86.5-95.6**	**3.1**	**0.27**	**16.8**
	qGY-10a	12LN	10	61.8	S10_6734839 - S10_8210492	50.8-63.1	3.1	−0.21	7.09
	qGY-10b	12NN, Comb-NN	10	121.3	S10_54423190 - S10_55477998	117.2-125.5	4.6	−0.54	12.9
TGW	qTGW-1	11LN	1	2.8	S1_1573765 - S1_5966723	0-12.3	3.3	1.19	17.6
	qTGW-3	11NN, Comb-LN	3	95.1	S3_54561579 - S3_56298715	92.4-98.8	3.7	−1.22	9.19
	qTGW-4	12NN	4	11.7	S4_4317922 - S4_5726443	10.6-16.4	3.1	−0.78	7.3
GS	qGS-1	11LN	1	36	S1_10813941 - S1_14302572	25.7-36.8	3.2	−0.08	8.11

Traits indicate *Chl1, Chl2, Chl3* chlorophyll contents at (vegetative, anthesis, and maturity) stages, *PH* plant height (cm), *AD* days to anthesis, *MC1*% stover moisture content, *MC2*% head moisture content, *BY* biomass yield (t. ha^−1^), *GY* grain yield (t. ha^−1^), *TGW* thousand grain weight (g), *GS* grain/stover ratio (%). QTL name indicates q for QTL followed by trait name to which the QTL was detected and by the chromosome number on which it was detected. Environments: 11NN, 12NN indicate that QTLs detected in normal N treatments in 2011 and 2012 years respectively; 11LN, 12LN indicate QTLs detected in low N treatments in 2011 and 2012 years respectively; Comb-NN, Comb-LN indicates QTLs detected on trait values averaged over two years (2011 and 2012) in normal N and low N treatments respectively. Chr, chromosome on which QTL was detected. ^a^2.0-LOD drop support interval of the QTL; ^b^Additive effect: positive values of the additive effect indicate that alleles from CK60 were in the direction of increasing the trait score and vice versa; ^c^Percentage of phenotypic variation explained by the QTL. If more than one QTL were detected on the same chromosome for a trait, QTLs identified were serially numbered. QTLs highlighted in bold are the validated QTLs, which were also detected in earlier study reported by Gelli et al. [46]

### QTLs for chlorophyll contents

For chlorophyll contents measured at three different stages of plant growth, six QTLs were detected on chromosomes 2, 3, and 10 with LOD scores range from 3.1 to 4.9 and R^2^ values from 6.0 to 14% (Table 4). Pooled analysis of the data from two years detected QTLs for chlorophyll content at vegetative stage on chromosome 10 for Low N treatment (comb-LN). Individual year analysis detected QTLs for chlorophyll contents at vegetative and maturity stages under normal nitrogen only in 2012. QTLs for chlorophyll contents on chromosomes 3 and 10 were overlapped with biomass and grain yield QTLs (Fig. 1). No significant QTLs were detected for chlorophyll content measured at anthesis (Chl2) in this population.

### QTLs for morphological traits

Fourteen significant QTLs with LOD scores ranging from 3.0 to 5.7 explaining 7 to 18% of phenotypic variation were detected for four morphological traits. For plant height, five QTLs were detected on chromosomes 2, 3, 6, and 9 and most of them were found in 2011. The QTL on chromosome 3 was detected under LN in 2011 and 2012. This QTL was also detected in the pooled data from LN across two years (Table 4). For days to anthesis, *qAD-9* was detected on chromosome 9 with a LOD score of 5.7 under normal N condition. This QTL was also detected in the pooled data from two years under LN condition. This constitutive QTL explained 15% of the phenotypic variation and overlapped with plant height, head moisture content and grain yield QTLs (Fig. 1). The favorable allele is contributed by the CK60 parent. QTL mapping for individual years detected presence of QTLs for days to anthesis under normal N on chromosomes 1, 2 and 3. Two QTLs were found to control stover moisture content in this population on chromosomes 1 and 8. For the QTL (*qMC1-1*) detected under LN, allele from CK60 contributed positively to increase the stover moisture content. In contrast, for the other QTL detected under NN conditions on chromosome 8 (*qMC1-8*), positive allele from San Chi San increased the stover moisture content. Similarly, two QTLs controlling head moisture content (MC2) were detected on chromosome 1 and 9 under LN conditions. For *qMC2-9*, positive allele form CK60 increased the head moisture content and for the other QTL allele from San Chi San contributed for this trait.

### QTLs for yield related traits

For biomass yield, three QTLs were detected on chromosomes 3, 7, and 8 with LOD scores ranging from 3 to 4.1 with R^2^ from 6.8 to 11%. Of these, QTL on chromosome 7, *qBY-7* was detected under low N condition in 2011, also with the pooled data from two years (comb-LN). The QTL for biomass detected on chromosome 3 (*qBY-3*) under low N condition was overlapped with QTLs for chlorophyll content measured at vegetative stage detected under normal N in 2012. For grain yield, five significant QTLs explaining 6.6 to17% of the phenotypic variance were identified on chromosomes 2, 8, 9 and 10. Out of these, three QTLs were detected under normal N in 2012 and also in the comb-NN, this is a consistent QTL across years. A grain yield QTL on chromosome 9 (*qGY-9)* was detected in the pooled data under normal nitrogen, overlapped with QTLs for days to anthesis and plant height. This QTL explained 17% of the phenotypic variation. Three QTLs controlling thousand grain weight (*qTGW-1, qTGW-3* and *qTGW-4*) explaining 7.3 to 17.6% of the phenotypic variance were identified on chromosomes 1, 3, and 4. Of these, a QTL (*qTGW-3*) was identified in both nitrogen conditions. For two QTLs (*qTGW-3*, and *qTGW-4*) the positive allele from San Chi San increased thousand grain weight of the seed. But, for the other QTL, *qTGW-1*, allele from CK60 increased the trait. One QTL for grain-to-stover ratio (*qGS-1*), explaining 8.1% phenotypic variance was detected on chromosome 1 under LN in 2011. For this QTL, the allele from San Chi San increased the grain-to-stover ratio in the seed.

### Validation of QTLs across mapping populations

Validation of QTLs in different genetic backgrounds/environments is required before being used in marker assisted selections to rule out statistical errors [48]. Based on QTL confidence intervals, we compared the QTLs detected in this study with the QTLs detected in our earlier reported study [46] for N-stress tolerance, where CK60 was used as a common parent. Among the 32 QTLs identified in this study, nine major QTLs were overlapped with the chromosomal regions carrying the QTLs detected in our earlier study using CK60/China17 population (highlighted in Table 4 and Fig. 1). These validated QTLs were located on chromosomes 1, 6, 7, 8 and 9. The QTLs that were identified in both mapping populations are likely most reliable and stable QTLs across germplasm. These validated QTLs include: one major QTL each for days to anthesis and stover moisture content on chromosome 1 (*qAD-1b*, *qMC1-1*), one major QTL for plant height on chromosome 6 (*qPH-6*), one major QTL each for biomass yield on chromosome 7 and 8 (*qBY-7*, *qBY-8*) and one major QTL each for days to anthesis, grain moisture content, plant height and grain yield on chromosome 9 (*qAD-9*, *qMC2-9*, *qPH-9*, *qGY-9*) detected under different nitrogen levels. The overlapped QTLs with our earlier study using CK60/China17 RIL population were listed in Additional file 2. Of these nine validated QTLs, one major QTL explaining ~15% phenotypic variation for days to anthesis on chromosome 9 (*qAD-9)* was detected consistently under both NN and LN conditions in both mapping populations, which is a stable QTL across N-environments and populations.

### Comparison of QTL regions under contrasting N environments

In this study, 32 QTLs were identified using a SNP based genetic map in the RIL population tested under two contrasting nitrogen levels for two years. However, almost half of these QTLs were detected under one N level in each year, indicating that the traits were controlled by different genes under different N conditions. QTLs either detected under low N in one year and across two normal N (pooled average of two years under normal N) or detected under normal N in one year and across two low N conditions (pooled average of two years under low N) were considered as consistent across environments. A QTL detected in multiple environments is a relatively stable QTL and is important for plant breeding [82]. In this study, two QTLs (*qAD-9* for days to anthesis and *qTGW-3* for thousand grain weight) were detected across normal N and low N environments (Table 4), suggesting that they were relatively stable. These two QTLs contribute 9.2 to 15% of the total trait variation.

### Identification of candidate DEG transcripts for N-stress tolerance

To identify candidate DEG transcripts controlling agronomic traits under N-stress conditions, the DEG transcripts found between parents and/or RIL bulks were overlaid on to the validated QTL regions that are common between two mapping populations. Differential expression of gene transcripts between parents and RIL bulks were calculated from the transcriptomes (RNA-seq data) generated earlier [65] on the root tissues of different sorghum genotypes grown under N-stress. False discovery rate (FDR) ≤ 0.001 and the absolute value of |log_2_ (Fold change) | ≥ 1 were used as thresholds to judge the significance of differences in transcript abundance. The RNA-seq results showed 486 DEG transcripts between parents (CK60 vs San Chi San, Additional file 3), and all of these DEG transcripts observed between parents may not be responsible for the difference in N-stress tolerance. Therefore, we analyzed the transcriptome profiles of bulked RNA extracted from five best and worst performing RILs of CK60/San Chi San population selected based on the biomass yield under N-stress to normalize the background noise of DEG transcripts not related to the N-stress tolerance. A total of 131 transcripts were found to be differentially expressed between RIL bulks with high and low NUE (Additional file 4). Of these, 54 DEG transcripts were common between parents and RILs (Additional file 5). Some of these DEG transcripts were flanked by QTL intervals and are involved in some metabolic pathways. The first class of gene transcripts include, the genes involved in nitrogen metabolism and utilization, such as nitrate transporter (NRT1, NRT 2.4), lysine histidine transporter, nitrite reductase (NiR), NOD26 and early nodulin gene. Expression level of this class of gene transcripts associated with the N-metabolism and will affect the biomass and grain yield of the plant. Second class of gene transcripts were involved in low-nitrogen stress responses, mainly abiotic stress response genes, phytohormone signal response genes including cytokinin response regulator and auxin binding protein. Next class of gene transcripts were involved in translocation and senescence-related proteins such as amino acid permease, signaling proteins like MADs box transcription factors, environmental adaptation and stress related proteins like lectin protein kinase genes. To narrow down the list of candidate DEG transcripts associated with N-stress tolerance, we focused on DEG transcripts located in the genomic regions on chromosomes 1,6,7,8, and 9 of sorghum where QTLs were validated across two mapping populations and also co-localized with QTLs for other traits reported so far. These DEGs transcripts will be considered as candidate DEG transcripts associated with the QTLs of NUE for the future prospects (Tables 5 and 6). These candidate gene transcripts will lead to a thorough understanding of physiological significances of the genes associated with NUE in sorghum.

**Table 5:** List of DEG transcripts between CK60 vs San Chi San associated with validated QTL confidence intervals detected using RNA-seq

Gene id (v1.4)	Chr	Start	logFC	Annotation
Sb01g032720	1	55,657,279	4.48	senescence-related gene 1
Sb01g032990	1	56,004,806	−6.49	3beta-hydroxysteroid-dehydrogenase/decarboxylase isoform 2
Sb01g033010	1	56,047,918	9.15	UB-like protease 1A
Sb01g033090	1	56,202,769	4.51	Mannose-binding lectin superfamily protein
Sb01g033360	1	56,595,053	−5.01	acetoacetyl-CoA thiolase 2
Sb01g033510	1	56,844,396	−3.37	RING/U-box superfamily protein
Sb01g035040	1	58,570,198	3.77	annexin 3
Sb01g035710	1	59,334,810	8.66	F-box/RNI-like/FBD-like domains-containing protein
Sb01g035910	1	59,529,076	9.33	glutathione S-transferase THETA 3
Sb01g036330	1	59,936,853	−2.75	Ribosomal protein L16p/L10e family protein
Sb01g037560	1	61,109,734	3.98	Mitochondrial import inner membrane translocase subunit Tim17/Tim22/Tim23 family protein
Sb01g037730	1	61,307,485	−2.94	
Sb01g037940	1	61,514,738	7.80	PYR1-like 6
Sb01g038720	1	62,214,256	−7.04	lysine histidine transporter 1
Sb01g039370	1	62,800,020	5.56	Ankyrin repeat family protein
Sb01g039390	1	62,807,357	−6.51	heat shock cognate protein 70-1
Sb01g041180	1	64,497,962	−5.30	heat shock protein 21
Sb06g014250	6	39,313,831	4.94	multidrug resistance-associated protein 9
Sb06g014400	6	39,867,816	−4.42	heat shock protein 70
Sb06g014450	6	39,970,615	3.70	FAD-binding Berberine family protein
Sb06g014550	6	40,216,040	3.34	senescence-related gene 1
Sb06g015520	6	43,082,617	8.33	B-block binding subunit of TFIIIC
Sb06g016020	6	44,422,691	2.67	
Sb06g016043	6	44,460,095	7.30	mediator subunit 8
Sb06g016160	6	44,576,681	2.55	seed storage 2S albumin superfamily protein
Sb06g016570	6	45,400,235	10.55	tetraspanin3
Sb07g021940	7	56,219,220	3.00	PEP1 receptor 1
Sb07g022320	7	56,757,993	5.98	Cytochrome P450 superfamily protein
Sb07g023140	7	57,977,647	5.21	Gibberellin receptor GID1L2
Sb07g023220	7	58,087,984	−3.46	phospholipase A 2A
Sb07g023260	7	58,157,388	−8.65	tetraspanin9
Sb07g023750	7	58,708,629	11.2	
Sb07g023770	7	58,722,654	11.2	rotamase cyclophilin 5
Sb07g024200	7	59,189,842	−7.60	Ribosomal protein L1p/L10e family
Sb07g026735	7	61,850,599	−5.10	BTB-POZ and MATH domain 1
Sb07g026825	7	61,987,674	−4.46	Pyridoxamine 5\’-phosphate oxidase family protein
Sb08g002210	8	2,325,578	7.19	Protein of unknown function (DUF567)
Sb08g002590	8	2,673,615	−2.69	WRKY DNA-binding protein 55
Sb08g003156	8	3,465,443	5.29	
Sb08g003850	8	4,444,512	7.37	RING/FYVE/PHD zinc finger superfamily protein
Sb09g018750	9	47,019,339	3.26	Glycosyl hydrolase superfamily protein
Sb09g019880	9	48,871,032	7.33	Microtubule associated protein (MAP65/ASE1) family protein
Sb09g020980	9	50,414,518	9.79	Peroxidase superfamily protein
Sb09g021000	9	50,425,845	3.75	Peroxidase superfamily protein
Sb09g021720	9	51,194,456	−2.89	histone deacetylase 8
Sb09g022390	9	52,044,973	8.36	Ribosomal protein S8 family protein
Sb09g022400	9	52,083,991	−4.80	cytochrome-c oxidases; electron carriers
Sb09g022580	9	52,235,414	−2.69	heat shock protein 70
Sb09g025840	9	55,253,011	−3.06	Protein of unknown function (DUF506)
Sb09g025900	9	55,284,480	−3.25	heat shock protein 101
Sb09g026440	9	55,716,410	−2.64	winged-helix DNA-binding transcription factor family protein
Sb09g027380	9	56,449,825	−3.15	Serine, threonine protein kinase, STT7 homolog STN7
Sb09g027470	9	56,561,299	5.29	Disease resistance protein (TIR-NBS-LRR class) family
Sb09g028960	9	57,721,281	4.34	ribosomal protein, L13
Sb09g029640	9	58,275,799	−4.87	Rad21/Rec8-like family protein

Chr, chromosome number; Log_2_ ratio; number of folds the gene is differentially expressed in RNA-seq. Positive sign indicates gene transcript expressed high in CK60

ns, indicate the gene is not differentially expressed between CK60 and San Chi San

**Table 6:** List of DEG transcripts between bulks of RILs with low vs high NUE associated with validated QTL confidence intervals detected using RNA-seq

Gene id (v1.4)	Chr	Start	logFC	Annotation
Sb01g033980	1	57,393,170	7.57	Ribosomal protein S4
Sb01g034150	1	57,603,650	1.56	Amino acid permease family protein
Sb01g034700	1	58,128,360	1.36	terpene synthase 21
Sb01g038720	1	62,214,256	−4.04	lysine histidine transporter 1
Sb01g039690	1	63,184,945	2.5	laccase 17, nitrite reductase (NiR)
Sb06g014550	6	40,216,040	1.80	senescence-related gene 1
Sb06g015880	6	43,936,495	1.51	Xyloglucan endotransglucosylase/hydrolase family protein
Sb06g018490	6	48,086,361	2.40	UDP-glucosyl transferase 85A2
Sb07g023600	7	58,479,475	5.86	
Sb07g023602	7	58,483,111	7.10	
Sb07g023605	7	58,484,711	8.09	
Sb07g028110	7	63,104,949	1.52	Calcium-binding EF-hand family protein
Sb08g001780	8	1,755,888	1.43	early nodulin-like protein 18
Sb08g003110	8	3,380,820	2.40	cytochrome P450, family 94, subfamily C, polypeptide 1
Sb08g006800	8	11,128,291	1.92	receptor like protein 7
Sb08g015850	8	42,032,993	−4.74	Zinc finger C-×8-C-×5-C-×3-H type family protein
Sb09g018750	9	47,019,339	2.77	Glycosyl hydrolase superfamily protein
Sb09g020980	9	50,414,518	2.47	Peroxidase superfamily protein
Sb09g021000	9	50,425,845	2.11	Peroxidase superfamily protein
Sb09g023910	9	53,527,963	6.13	Nucleic acid-binding, OB-fold-like protein

Chr, chromosome number; Log_2_ ratio; number of folds the gene is differentially expressed in RNA-seq. Positive sign indicates gene transcript expressed high in low NUE RIL bulk

ns, indicate the gene is not differentially expressed between low and high NUE RIL bulks

## Discussion

Over the past half century, use of the nitrogen (N) fertilizers has markedly increased crop yields, but with considerable negative effects on environment and human health. Consequently, there has been a strong push to reduce the amount of N fertilizer used by maximizing the nitrogen use efficiency (NUE) of crops. Different approaches have been targeted to improve the NUE of crop plants. One such approach would be to use classical genetics to improve the NUE of a crop plants, involves conventional breeding and QTL mapping in combination with marker-assisted selection to track the key regions of the chromosome that segregate for NUE. Another approach would be characterizing the NUE-associated genes which co-segregate with QTLs for NUE traits, and use the profiles of specific genes to combine with plant physiology and genetics to improve plant performance under N-limited conditions. However, QTLs and the candidate genes that segregate with the detected QTLs in one mapping population may not be the same with those detected in other populations. Validation of QTLs and associated candidate genes across mapping populations is critical for finding stable QTLs and common genes to target for improved NUE of crop plants through marker assisted selection.

### Co-localization of validated QTLs across mapping populations and candidate genes co-segregate with QTLs for N-stress tolerance

Breeding varieties/hybrids with improved NUE is one of the approaches for sustainable sorghum productivity in nitrogen-limited areas. Genetic improvement of NUE is challenging because it is a quantitative trait and its molecular basis is inherently complex. Therefore, it is essential to understand the genetic architecture of NUE traits for genetic manipulation of NUE through marker assisted selection. In sorghum, significant positive correlation between traits have been reported and QTLs for correlated traits are known to be mapped together [83–85]. Co-mapping of QTLs for correlated traits may result from either tight linkage of several genes [86] or the pleiotropic effect of major genes [87]. Co-mapping or co-localization of QTLs is therefore important as it provides a clue on the interpretation of the relationships among such traits [88], and can assist breeders in identifying the best QTL alleles for manipulating multiple traits simultaneously in marker assisted breeding.

QTLs for different traits were declared co-incident/co-localized when the QTL confidence intervals were over-lapping. For example, Mace et al. [89] did a comprehensive analysis and projected 771 QTLs relating to 161 unique traits tested under different environmental conditions from 44 studies onto the sorghum consensus map. Similarly, the nine validated QTLs found in this study on chromosomes 1, 6, 7, 8, and 9 between two of our mapping populations for N-stress tolerance were compared with the QTLs reported in sorghum for other traits by overlaying the physical positions of markers on V1.4 sorghum genome to determine the co-localization of common QTLs across germplasm. The co-localized regions for example, a cluster of two QTLs on chromosome 1 were detected in this study using CK60/San Chi San population for days to anthesis (*qAD-1b)* and stover moisture content (*qMC1-1*) under LN. These QTLs were co-localized with QTLs detected in our earlier study using CK60/China17 RIL population [46] for biomass yield detected under normal N, and chlorophyll content at flowering, thousand grain weight, grain to stover ratio under low N and days to anthesis, and head moisture contents were detected under both nitrogen treatments (Additional file 2). Besides, this co-localized region harbors QTLs for stay-green, conditioned by *Ma3* gene encoding phytochrome B, involved in photoperiod sensitivity were reported earlier [52]. Similarly, QTLs for green leaf area at maturity [83], days to anthesis [83, 90], plant architecture [91], and fresh panicle weight and plant height [92, 93] were also reported in this region. Also, by overlying of DEG transcripts from RNA-seq data on to this co-localized region, we detected candidate DEG transcripts between parents (CK60 vs. San Chi San) and RIL bulks (high vs. low NUE RIL bulk) (Tables 5, and 6). Of these detected DEG transcripts, seven were overlapped with the DEG transcripts detected in our earlier study using CK60/China17 population [46], and had higher expression levels in consistent with either CK60 or China17 parents. Among these DEG transcripts, *Lysine histidine transporter 1* (LHT1) transcript was differentially expressed between parents and RIL bulks. LHT1 was massively expressed in San Chi San and high NUE RIL bulk, similar to China17 which was reported earlier [46]. High affinity amino acid transporter (LHT1) is an amino acid permease homolog and was reported to be expressed in roots and responsible for uptake of amino acids from soil into the roots [94] and distributes to shoots through xylem [95] for further metabolism under N-stress. Arabidopsis seedlings deficient in LHT1 fail to use Glu or Asp as a nitrogen source because of the severe inhibition of amino acid uptake, and *lht1* mutants show growth defects on fertilized soil and were rescued with LHT1 re-expression in green tissue. LHT1 overexpression let to a several fold increase in capacity for amino acid uptake in roots. This suggests LHT1 overexpression may improve the N-efficiency of plant growth under N-stress [94]. Another DEG transcript associated with *qAD-1* was amino acid permease (Table 6). Ectopic expression of *Vicia faba* amino acid permease in peas increased the seed sink strength for nitrogen, amino acids and improved plant nitrogen status and seed size by 20-30% with higher seed protein content [96]. Transcript encoding Glutathione-S-transferase gene was abundant in sensitive genotype, CK60 under N stress, similar results were also found under cold stress [67]. Glutathione S-transferases (GSTs) involved in detoxification of xenobiotic compounds and oxygen radicals [97] and are useful markers in the detection of stress in plant metabolism. Reactive oxygen species are produced under abiotic stress, which damage cellular membranes and eventually cell death. It is likely that high abundance of GSTs may protect sorghum cells from oxidative stress that is prominent in N-stress sensitive genotypes.

A major QTL affecting plant height (*qPH-6*) explaining ~17% of phenotypic variance was detected on chromosome 6 under NN conditions in this study using CK60/San Chi San population. Positive allele from San Chi San increased the plant height by 7 cm. This region was co-localized with a genomic region containing QTLs for plant height and grain yield detected under low N in our earlier study using CK60/China17 population [46] (Additional file 2). In this co-localized region, QTL clusters for plant height [90, 92], panicle architecture [91], kernel weight [90], biomass yield [92], green leaf area at maturity, panicle length, grain yield, seed weight and a major QTL for plant height, *QPhe-sbi06-1*, conditioned by the *Dw2* gene [83] were also reported earlier. The *Dw2* locus was reported to be genetically linked to the major maturity locus, *Ma1* and explained 55% of the variation in plant height [98–100]. Overlying of the DEG transcripts on to this co-localized region between CK60 vs San Chi San and RIL bulks with high vs low NUE found candidate DEG transcripts associated with this plant height QTL, *qPH-6* (Tables 5, 6). These candidate DEG transcripts including seed storage 2S albumin, TFIIIC, HSP 70 and multidrug resistance-associated protein-9 and which were overlapped with the candidate DEG transcripts detected in our earlier study using CK60/China17 population [46], and had higher expression levels in consistent with either CK60 or China17. Transcripts encoding TFIIIC and seed storage 2S albumin were expressed higher in CK60 and senescence-related gene 1 was expressed higher in CK60 and in RIL bulk with low NUE. However, HSP70 had a higher expression in San Chi San similar to China17 [46]. In addition, a *DW*_*2*_ transcript encoding multidrug resistance-associated protein-9 homolog showed higher transcript abundance in CK60, similar finding was observed in earlier study [46] and indicates this common DEG transcript may be involved in regulating plant height under N-stress in the seedlings (Table 5). Another candidate DEG transcript encoding mediator subunit 8 associated with *qPH-6*, was abundant in sensitive genotype CK60 under N-stress. In Arabidopsis, Mediator complex subunit 8 was reported to regulate the organ size and *Atmed8* mutant plants showed delayed flowering in both short and long days and had smaller flowers compared to wild type plants as a result of reduced cell expansion [101]. Mediator subunits, MED25 and MED8 were involved in the production of root hairs in Arabidopsis [102, 103]. The absence of root hairs in *Atmed25* and *Atmed8* was due to inappropriate distribution of hydrogen peroxide (H_2_O_2_) and superoxides (O_2_^−^) on the surface of tap roots.

Similarly, a major QTL for biomass yield (*qBY-7*) was detected on chromosome 7 under LN conditions explaining 11% of phenotypic variance in CK60/San Chi San population. The positive allele from high yielding parent San Chi San, similar to China17 [46], increases biomass yield by 1.2 t. ha^−1^. This region is co-localized with major QTLs for biomass yield and chlorophyll contents detected under contrasting nitrogen conditions reported in our earlier study using CK60/China17 population [46] (Additional file 2). This region was also co-localized with the region containing QTL for stay-green [52], plant height and panicle length [83, 85], fresh total biomass yield and dry total biomass yield [104], and panicle architecture [90, 98] reported earlier. This major biomass yield QTL corresponds to a major plant height gene, *DW3* (Sb07g0232730). *Dw3* is a homologue of maize *Br2* and Arabidopsis *PGP1*, and encodes a protein similar to ATP-binding cassette transporters of the multidrug resistant class of P-glycoproteins [105]. *Dw3* is known to result in reduced grain yield in sorghum, with pleiotropic effects on the number of kernels per panicle and kernel weight, tiller number and panicle size [106, 107]. *Dw3* reduces grain yield mainly through reduced stem mass, and grain size but not the actual grain number [108]. Overlying of the DEG transcripts between parents and RIL bulks on to this co-localized region found candidate DEG transcripts associated with QTL, *qBY-7* (Tables 5, 6). Of these, Gibberellin receptor GID1L2 and rotamase transcripts were abundant in CK60 and phospholipase A and ribosomal proteins L1p/L10e were abundant in San Chi San similar to China17 from earlier study [46]. On chromosome 8, a major QTL for biomass yield was detected under LN similar to earlier studies. This co-localized region containing a DEG transcript encoding Early Nodulin gene (ENOD), had higher expression in RIL bulk with high NUE. ENOD was reported to effect increased total amino acids and N as well as dry biomass and seed yield. Transgenic rice plants over-expressing the *OsENOD93-1* gene had increased shoot dry biomass and seed yield. *OsENOD93-1* gene was shown to express high levels in roots and higher concentration of amino acids in xylem sap was detected in transgenic plants especially under N stress [109].

On chromosome 9, a QTL controlling days to anthesis (*qAD-9*) was detected consistently across all the environments, and a QTL for grain moisture content (*qMC2-9*) detected under LN, plant height and grain yield were detected under NN conditions in this study. In the corresponding region, a QTL cluster containing QTLs for chlorophyll content measured at flowering and maturity, days to anthesis detected under both N regimes, plant height, total biomass and grain yield were detected under normal N regime in our earlier study (Additional file 2) [46]. In this co-localized region, QTL for flowering time [90, 100], total seed weight [91], stay green [110, 111] and plant height [93] were detected earlier. In addition, a plant height QTL *(Sb-HT9-1)* was fine mapped to a ~ 100 kb region through association mapping [112], both *DW3* and *Sb-HT9-*1were consistently detected as the most important loci controlling plant height in crosses between tall and dwarf sorghum. In this region, our RNA-seq data detected DEG transcripts between CK60 vs. San Chi San and RIL bulks with high vs. low NUE (Tables 5, and 6). Of these, histone deacetylase 8, HSP 101 and STN7 transcripts were abundant in San Chi San and transcripts encoding ribosomal proteins S8 and L13, disease resistance proteins were abundant in CK60 similar to the earlier reported study [46]. The DEG transcript encoding Ser/Thr kinase (STN7) expression was up-regulated in root cells of rice under low-nitrogen stress [113]. These candidate DEG transcripts may be helpful in further understanding of the genetic basis of NUE and may also enrich available gene resources for breeding of high-NUE varieties of sorghum.

## Conclusions

Genetic markers for quantitative traits that are commonly identified in mapping populations will enhance selection for cultivar improvement. However, plant breeding community recognizes the necessity to validate these putative QTLs across various genetic backgrounds before embarking upon marker-assisted selection. In this study, we mapped and validated the QTLs detected for agronomic traits tested under contrasting N-levels in CK60/San Chi San population with the QTLs reported in our earlier study using another population, where CK60 was a common parent. These validated, common QTLs were considered as stable QTLs, may indicate the presence of major loci controlling the traits. Molecular markers flanking these common QTLs would be helpful in forward breeding to improve agronomic traits under N-stress. In addition, Illumina RNA-seq allowed to detect differential expression of common gene transcripts in the pleiotropic QTLs. However, these common DEG transcripts need to be characterized further by designing KASPar assays using the sequences of DEG transcripts and validate these markers on RIL mapping populations to check if the marker is segregating with the phenotype/trait. Identification of the candidate genes that affect a trait facilitate tracking the trait with markers through marker assisted breeding or clone the allele. Manipulating one or more of these gene products is expected to potentially increase the NUE of crops by further understanding the genetic components that contribute to these processes.

## Additional files

Additional file 1: Genetic distribution of SNPs discovered using genotyping-by-sequencing (GBS) in CK60/San Chi San RIL population. (XLSX 31 kb)

Additional file 2: List of QTLs detected in CK60/China17 population that are overlapped with QTL intervals of CK60/San Chi San population. (XLSX 13 kb)

Additional file 3: The list of differentially expressed gene transcripts identified between CK60 and San Chi San using RNA-seq. (XLSX 61 kb)

Additional file 4: The list of differentially expressed gene transcripts identified between RIL bulks with high and low NUE using RNA-seq. (XLS 65 kb)

Additional file 5: The list of common differentially expressed gene transcripts identified between CK60 and San Chi San, RIL bulks with high and low NUE using RNA-seq. (XLS 48 kb)

## Abbreviations

AD: Days to anthesis (days)
ANOVA: Analysis of variance
BY: Biomass yield (t.ha^−1^)
Chl1: Chlorophyll content at vegetative stage
Chl2: Chlorophyll content at anthesis
Chl3: Chlorophyll content at maturity
DEG: Differentially expressed gene
ENOD: Early nodulin gene
FDR`: False discovery rate
GBS: Genotyping-By-Sequencing
GS: Grain/stover ratio (%)
GST: Glutathione S-transferase
GY: Grain yield (t.ha^−1^)
h^2^: Narrow sense heritability
HSP: Heat shock protein
IciMapping: Inclusive composite interval mapping
LHT1: Lysine histidine transporter 1
LN: Low Nitrogen
LOD: Logarithm of odds
MAS: Marker assisted selection
MC1: Stover moisture content (%)
MC2: Head moisture content (%)
NN: Normal nitrogen
NUE: Nitrogen use efficiency
PH: Plant height (cm)
QTL: Quantitative trait locus
RILs: Recombinant inbred lines
SNPs: Single nucleotide polymorphisms
TGW: Thousand grain weight (g)
